# Supplementary figures and images for: Alterations of aqueous humor Aβ levels in Aβ-infused and transgenic mouse models of Alzheimer disease
Source: PLoS One. 2020 Jan 10;15(1):e0227618. doi: 10.1371/journal.pone.0227618 (PMC6953883; doi:10.1371/journal.pone.0227618)

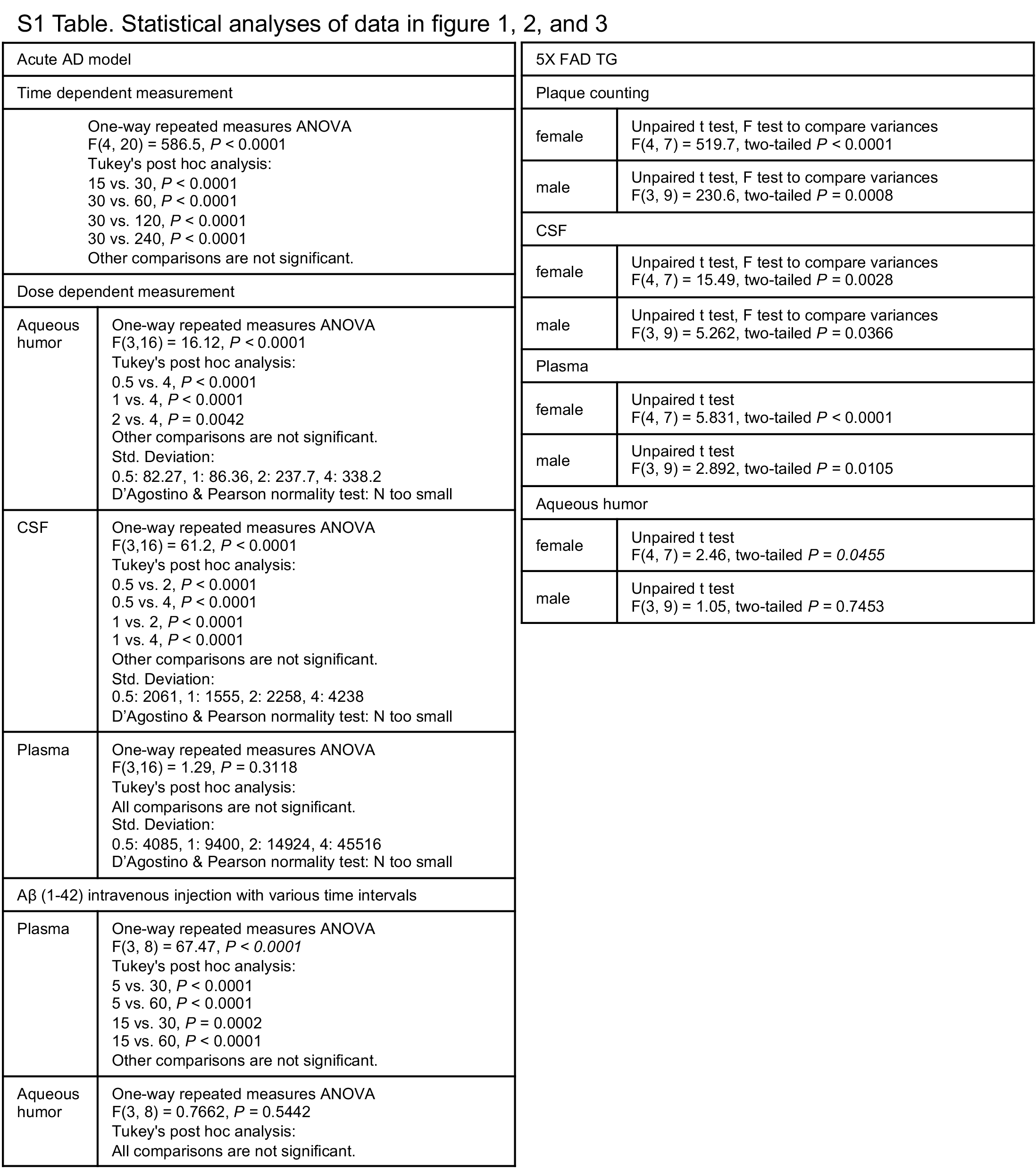

Supplement: S1 Table — (TIF) [file pone.0227618.s001.tif]
