# Supplementary material for: Alterations of aqueous humor Aβ levels in Aβ-infused and transgenic mouse models of Alzheimer disease
Source: PLoS One. 2020 Jan 10;15(1):e0227618. doi: 10.1371/journal.pone.0227618 (PMC6953883; doi:10.1371/journal.pone.0227618)
Supplement: Attachment — Submitted filename: Response to Reviewers.docx [file pone.0227618.s002.docx]

Dear Prof. Madepalli K. Lakshmana, Academic Editor of *PLOS One*,

Please find enclosed the revised manuscript, PONE-D-19-24249, newly entitled “**Alterations of aqueous humor Aβ levels in Aβ-infused and transgenic mouse models of Alzheimer disease**” by Kwak, Ko et al., that we are submitting for possible publication as a research article in *PLOS ONE*. In our previous version of the manuscript, the title was “**Correlations of amyloid-β levels between CSF and aqueous humor in transgenic and acute Alzheimer disease mouse models**”. We appreciate this opportunity to revise our manuscript, and we also appreciate for time and effort of you and all reviewers to provide critical and constructive comments. We ensured that the revised manuscript complied with the guideline of *PLOS One*.

Here, we provide a detailed point-by-point response to the reviewers’ comments below. The font styles are following:

- *Reviewer’s comments are in black italic.*
- Authors’ responses are in blue.
- Sentences from original manuscript are in gray highlighted.
- Sentences from revised manuscript are in yellow highlighted.

We hope our rework meets your requirements.

***RESPONSE TO THE REVIEWER’S COMMENTS:***

Reviewer #1: Synopsis

*1) in 230-232, as this sentence gives a generalized expression rather than what the authors can claim in this particular experiment. Rephrase perhaps as follows: “This specific animal model showed that ICV injected Aβ reaches the AHF fluid”. You cannot rule out other sources at all as the ICR animals are not TG and your Aβ ELISA is human specific. Even if the animals were TG the experiment could not rule out peripheral sources by mere ICV injection and concentration changes, since even in TG there should be a pre-established equilibrium and ICV Aβ injection just add to this level.*
[Reply] We appreciate the reviewer’s point. We have revised the sentence as below;

(Line 230) This specific animal model ruled out the other possible origins of Aβ detected in aqueous humor such as peripheral amyloid precursor protein cleavages, as the Aβ levels identified from the mouse will only be from those injected intentionally to the CSF region.

(Line 279) This acute AD model showed that ICV injected Aβ reaches the aqueous humor by excluding the other possible origins of Aβ detected in aqueous humor.

*2) Also the conclusion in line 240-241 has to be carefully revised as it is, it is greatly misgiving. If you inject Aβ in circulation and then measure it in AHF vs CSF, you could perhaps make such a conclusion but as it is this claim is just one of several possibilities. In addition, such an information is useless (in the context of your manuscript) unless you can show that it is accumulated in AHF. Otherwise, why measure Aβ in AHF or CSF (for that matter) if you can measure it in plasma?*
[Reply] Thank you for the critical insights. To make the transport route of Aβ from CSF to aqueous humor, we added a new set of experiments injecting Aβ into the tail vein and observe Aβ levels in aqueous humor. We added the result in the revised manuscript.

(Line 242) Thus, it is predictable that the Aβ monomer passes blood-brain barrier and ultimately enters the aqueous humor.

(Line 211) To investigate the migration route of Aβ, we injected the synthetic Aβ(1-42) intravenously into the tail vein and collected aqueous humor samples with various time intervals (5, 15, 30, and 60 minutes) since the injection. As a result, we observed that the Aβ(1-42) levels in blood plasma significantly decreased over the time, which is consistent to the previous plasma and serum stability study (**Fig 2F**). Aβ(1-42) levels in aqueous humor also decreased in the time-dependent manner which is similar to the blood plasma result. However, the progression was slower and Aβ(1-42) were highly detected in aqueous humor of 5 and 15 minutes group compared to the blood case of 30 and 60 minutes group (**Fig 2G**).

*3) The sentence in line 242-244 (“It is assured … TG mice”) is also not correct/justified in the present study. CSF Aβ is a biomarker of AD because of its altered levels in AD vs control and its inverse association with the amount of Aβ deposits in the brain but not merely because Aβ can be measured in CSF (as control subjects have much higher levels of Aβ in the CSF than AD).*
[Reply] We agree with your important comment. In the revised manuscript, we compared CSF Aβ levels, aqueous humor Aβ levels, blood plasma Aβ levels, and amyloid deposition levels in the brain tissue of 5XFAD AD transgenic mice. We removed the sentence and hoped that the removal clarifies the points we attempted to make.

(Line 242) It is assured that aqueous humor Aβ can be a surrogate biomarker for the amyloid plaque burden in the brain through comparing WT mice with TG mice.

*4) Also consider the following shortcoming of the study: You might have been able to provide some clues if you had measured changes in AHF Aβ in relation to CSF Aβ and the Aβ deposits in the brain of the TG animal to establish that levels of Aβ in AHF like that in CSF reflect the extend of Aβ deposits in the brain. Thus currently, the Fig 1 results does not really say much as the comparison between TG vs WT has limited value since your Aβ ELISA can only measure human Aβ. The main interesting point in Fig. 1 is the Aβ level at 12 vs 24 months but since you do not show the levels of plaques and the levels of Aβ in CSF you cannot establish a proper relationship to point out AHF Aβ as a surrogate biomarker.*

[Reply] Thank you for your suggestion. As the given revision time was limited to prepare aged mice, we could not expand our study with APP/PS1 mice. Instead, we replaced and expanded our transgenic mouse study with the 5XFAD mouse model that is reported to show faster pathological development than APP/PS1. And we were lucky to have these mice in both genders in adult and aged groups; 3.7-month-old (n = 5) for adult female mice, 14-month-old (n = 8) for aged female mice, 5-month-old (n = 5) for adult male mice, and 12-month-old (n = 10) for aged male mice. In this revised manuscript, we compared CSF Aβ levels, aqueous humor Aβ levels, blood plasma Aβ levels, and amyloid deposition levels in figure 3.

*5)- In Abstract, line 26-28. The author claimed that they “… investigated whether the Aβ in the aqueous humor in AD models is directly correlated with the misfolding protein of the central nervous system.” You did not do such an investigation in this study as far as I can deduct from your experiment. As a matter of fact your ICV model reject your statement as you cannot claim (and it is very unlikely) that the synthetic Aβ monomers were all misfolded (not to mention that even in TG or human, one cannot claim that the soluble Aβ peptides are misfolded even though many believe so mainly because of confusion that aggregation can only occur due to misfolding of Aβ peptides and neglect that aggregation may also occur simply because of an excessive accumulation in e.g. parenchymal fluid in the brain.*

[Reply] We apologize for our misleading in the abstract. In the revised manuscript, we omitted this issue by rewriting the entire abstract. The issued sentence is now removed.

(Line 26) we investigated whether the Aβ in the aqueous humor in AD models is directly correlated with the misfolding protein of the central nervous system.

*Other issues:

1. ICR (mice) is not defined in abstract or the rest of the text.*

[Reply] Thank you for your elaborate point. We have included the definition of ICR.

(Line 80) ICR mice

(Line 100) Institute of Cancer Research (ICR) mice (strain name; Crl:CD1)

*2. Is the Age-dependent increase in AHF Aβ significant? Provide p-values!*

[Reply] We newly conducted the experiments comparing adult and aged transgenic male and female mice. AHF showed the age-dependent increase only in female group. We marked the significance with the p-value (=0.0455) in figure 3 and S1 table.

*3. Why didn’t you measure Aβ in serum, CSF and brain extracts of dTG?*

[Reply] Thank you very much for the suggestion. In the revised manuscript, we report analyses of Aβ alterations in blood, CSF, brain tissues, and aqueous humor of transgenic mice in figure 3.

*4. To ascertain route of Aβ to AHF, why not also inject Aβ iv in circulation, which is much easier to do and would provide additional information?*

[Reply] Thank you for your suggestion. In revision, we have explored the path of Aβ by the intravenous injection of Aβ in figure 2*.* We believe this new experiment made our evidence stronger.

(Line 211) To investigate the migration route of Aβ, we injected the synthetic Aβ(1-42) intravenously into the tail vein and collected aqueous humor samples with various time intervals (5, 15, 30, and 60 minutes) since the injection. As a results, we observed that the Aβ(1-42) levels in blood plasma significantly decreased over the time, which is consistent to the previous plasma and serum stability study (**Fig 2F**). Aβ(1-42) levels in aqueous humor also decreased in the similar time-dependent manner pattern to the blood plasma result. However, the progression was slower and Aβ(1-42) were highly detected in aqueous humor of 5 and 15 minutes group compared to the blood case of 30 and 60 minutes group (**Fig 2G**).

*5. In Fig 3, plasma Aβ42 at 4 nmole ICV injection is missing.*

[Reply] Thank you for your elaborate point. We performed the experiment again and analyzed the Aβ(1*-*42) level in plasma of each ICV injection (0.5, 1 , 2, and 4 nmol) in figure 2.

*6. In Fig 3, it is difficult to appreciate the importance/significant of the comparisons with the highest conc group (i.e. 4 nmol group), particularly since there is no difference between 0.5 vs 1 nmol-groups or 2 vs 4 nmol groups (in 3B)? It is important here that direct correlation graphs plus their r- and p-values are shown, as well.*

[Reply] Thank you for your important suggestion. We added the scattering plot graph in figure 2, where each plot simultaneously shows the Aβ(1-42) level of CSF and aqueous humor.

Reviewer #2: Reviewer Comments to Editor:
Recommendation: Accept after major revision.

*Abstract:
• Well-organized and well written.*

[Reply] Thank you for your compliment.

*• Since the word limit is 300, I would suggest authors to include few sentences of background information.*

[Reply] We appreciate for your suggestion. We have included a general description of Alzheimer’s disease.

(Line 23) Alzheimer’s disease (AD) is an ageing-related neurodegenerative disease characterized and diagnosed by deposition of insoluble amyloid-β (Aβ) plaques in the brain.

*• Preferable to be consistent with either Aβ (1-42) or amyloid beta.*

[Reply] We appreciate for your suggestion. Our newly revised manuscript uses Aβ(1-42) for all the experiments and Aβ only when we state backgrounds and previous studies.

*Introduction:
• In the first paragraph, few sentences are written without any citations. Citations would strengthen use point of emphasis.*

[Reply] We are very sorry for our mistake. We added new citations.

*• It is advisable to revise last paragraph where the author’s discuses about the experiment protocol instead of focusing on the importance of their study.*

[Reply] Thank you for the important suggestion. At the end of the results and the beginning of the discussion sections, we added a new sentence as below;

(Line 273) Collectively, in AD animal mice, we observed that (1) Aβ(1-42) monomers infused into CSF in the ICV region acutely transport to the blood and the aqueous humor, (2) artificially injected Aβ(1-42) levels in CSF are proportionally reflected in the aqueous humor in mice without human APP expression, and (3) the progressive decrease of Aβ(1-42) levels in representative fluid biomarkers, CSF and blood, is not observed in aqueous humor.

*• Please include one sentence showing the novelty and importance of your study.*

[Reply] We appreciate for your kind advice. We believe the same sentence we mentioned in the above reply shall deliver the message.

(Line 273) Collectively, in AD animal mice, we observed that (1) Aβ(1-42) monomers infused into CSF in the ICV region acutely transport to the blood and the aqueous humor, (2) artificially injected Aβ(1-42) levels in CSF are proportionally reflected in the aqueous humor in mice without human APP expression, and (3) the progressive decrease of Aβ(1-42) levels in representative fluid biomarkers, CSF and blood, is not observed in aqueous humor.

*Materials and Methods:
• In experiment 2, please elaborate how the aqueous humor at different time point from the same mice were collected.*
[Reply] We are very sorry that the explanation about the method was misgiving. In the revised manuscript, this is clearly stated in the results section and figure 1A.

(Line 175) We prepared five groups of Aβ-infused mice (male, n = 3 per group) each for separate time points and, in 15, 30, 60, 120, and 240 minutes since the ICV injection, we collected aqueous humor samples.

*Results:
• The authors have used male transgenic mice in their experiments whereas several studies (refer to the journals: PMID: 30820070, PMID: 26987699, PMID: 20442496) have reported that the incidence of AD is higher in females. So, I would suggest to repeat the experiments in female mice model to make the data more authenticate to the clinical data.*
[Reply] Thank you for your suggestion. As the given revision time was limited to prepare aged mice, we could not expand our study with APP/PS1 mice. Instead, we replaced and expanded our transgenic mouse study with the 5XFAD mouse model that is reported to show faster pathological development than APP/PS1. And we were lucky to have these mice in both genders in adult and aged groups; 3.7-month-old (n = 5) for adult female mice, 14-month-old (n = 8) for aged female mice, 5-month-old (n = 5) for adult male mice, and 12-month-old (n = 10) for aged male mice. In this revised manuscript, we compared CSF Aβ levels, aqueous humor Aβ levels, blood plasma Aβ levels, and amyloid deposition levels in figure 3.

*• Since hyperphosphorylated tau has been found in both AD and Glaucoma, suggesting a possible pathophysiologic link between the two diseases (PMID: 26425322), but no attempt was made to quantify hyperphosphorylated tau in this study.*
[Reply] In this study, we decided to focus on Aβ only as the BBB efflux of Aβ is clearly reported while that of tau is unclear yet. Under this circumstance, we believe that tau acute injection into mice, tau transgenic mouse preparation, and tau analyses in CSF, blood, and aqueous humor is beyond the scope of this study.

*• The authors measured the Aβ (1–42) concentration in CSF by injecting different concentration of Aβ (1–42) into the ICV region of normal adult ICR mice by following their previous study. But, the result showed significant differences (around 5-folds higher in this study compare to the previous result) between two studies. Why this discrepancy.*
[Reply] Thank you for the critical insight. To analyze Aβ in CSF using the ELISA kit, high dilution is essential for the assay. Before the ELISA test using all biofluids, we did the preliminary test to obtain the appropriate diluting factor. We applied 1,000X dilution for CSF samples in this study instead of 100-400X dilution factor in the previous study. Here is the example of ELISA results performed in our lab with the different dilution factors (the same samples with the 3 different dilution factors, 50x, 100x, and 1500x).

| Dilutions factor | conc.(pg/mL) |
| --- | --- |
| 50x | 1083 |
|  | 2005 |
|  | 989 |
|  | 849 |
|  | 876 |
| 100x | 676 |
|  | 1077 |
|  | 668 |
|  | 480 |
|  | 603 |
| 1500x | 4565 |
|  | 4287 |
|  | 3883 |
|  | 4814 |
|  | 4381 |

*• In the description of figure 1 results, the authors claimed that human Aβ (1-42) was not found in the aqueous humor of WT mice, but in figure 1 bar data, it clearly showed the presence of some amount Aβ (1-42) in WT.*
[Reply] This data is close to the background noise level and to circumvent the confusing data, we have decided to delete the WT mice group. Also, endogenous Aβ(1-42) detection issue in the murine was previously reported. Teich AF, Patel M, Arancio O. A reliable way to detect endogenous murine beta-amyloid. PLOS One. 2013;8(2):e55647. [1]

*• The authors claimed that Aβ transport from the central nervous system to the eye is through blood. So, I would recommend to perform the experiment for Aβ (1–42) concentration in plasma also to see how much Aβ (1–42) transported to the aqueous humor through the blood.*
[Reply] To make the transport route of Aβ from CSF to aqueous humor, we added a new set of experiments injecting Aβ into the tail vein and observe Aβ levels in aqueous humor. We added the result in the revised manuscript.

(Line 211) To investigate the migration route of Aβ, we injected the synthetic Aβ(1-42) intravenously into the tail vein and collected aqueous humor samples with various time intervals (5, 15, 30, and 60 minutes) since the injection. As a result, we observed that the Aβ(1-42) levels in blood plasma significantly decreased over the time, which is consistent to the previous plasma and serum stability study (**Fig 2F**). Aβ(1-42) levels in aqueous humor also decreased in the time-dependent manner which is similar to the blood plasma result. However, the progression was slower and Aβ(1-42) were highly detected in aqueous humor of 5 and 15 minutes group compared to the blood case of 30 and 60 minutes group (**Fig 2G**).

*• In figure 2B, the authors measured the half-time of Aβ efflux from CSF to blood and their finding is consistent with the work done by Shibata M. et al. (2000). In addition, Shibata M. et al. (2000) also mentioned that there was also a slow, time-dependent retention of Aβ in brain parenchyma with a t1/2 of 164.5 minutes. But, figure 2B shows after 34.63 minutes, release of Aβ is suddenly stopped from the brain which is not consistent with the Shibata study, i.e. there is no time-dependent retention of Aβ in brain.*
[Reply] Thank you for the important comment and previous research results we missed to interpret. Different from our study which utilized Aβ(1-42) peptides, ICR mice, and the single ICV injection method, Shibata M. et al. (2000) used Aβ(1-40), C57BL/6 mice, and injection by an implanted cannular in their report. Plasma stability of Aβ(1-42) is reported to be shorter and that of Aβ(1-40) by Okereke et al, J Alzheimers Dis. 2009;16(2):277-85. We suspect these variations may have contributed the difference between ours and Shibata et al.’s results. Also, the Figure 2B, which is now Figure 1B in the revised manuscript reports Aβ(1-42) levels in aqueous humor instead of the blood studied in Shibata M. et al. (2000).

*• For figure 2B & 3C, the authors followed the same procedure (injecting monomeric Aβ (1–42) directly into the intracerebroventricular (ICV) region of normal adult mouse brains), used same concentration (4nmol), same time point (30 minutes). But, why two different Aβ (1-42) concentrations.*
[Reply] Thank you for the important comment. Since the original data is too minimal to be differentiated, dilution factor of the samples has a great impact on the results. We initially made aqueous humor diluted to 1/200 when designing time-dependent aqueous humor exam. But, on the following experiment, we had to produce aqueous humor diluted to 1/100, since the ELISA value was not detectable in the low concentration group when diluted to 1/200. Therefore, two peaks seemed to be different between the graphs.

(Line 144) In time-dependent measurements, aqueous humor samples were 200-fold diluted. To detect the differences in low injection concentrations, aqueous humor samples were diluted 100-fold in dose-dependent measurements.

*• More labels are necessary in Figs 2A & 3A.*
[Reply] We added more labels in these figures, of which numbers are now changed to Figure 1A and 2A due to major rewriting of the manuscript.

Figure 1A and 2A

*Discussion:
• The authors should discuss more about how the Aβ (1–42) is transported into the aqueous humor through blood-brain barrier.*
[Reply] Thank you for the critical insights. We included the below statement in the revised manuscript.

(Line 284) We inferred the possible migration route of Aβ was through the blood. The Aβ peptide generated in the brain can be released into blood by the low-density lipoprotein receptor-related protein. Aqueous humor is secreted from the ciliary body, the circulation of which produces aqueous humor by blood ultrafiltration. As injecting Aβ(1-42) to the normal ICR mice intravenously, spiked Aβ concentration was reflected in aqueous humor. We concluded that the Aβ monomer passes blood-brain barrier and ultimately enters the aqueous humor. The exact molecular mechanism of how Aβ that was originally in the CSF can be detected in the aqueous humor needs further investigation.

Reviewer #3: This is a generally well written and interesting paper about the significantly increased levels of Aβ in the aqueous humor of transgenic AD mouse model in comparison to WT, and the ability to measure acutely perturbated levels of CSF Aβ in both the plasma and the aqueous humor of ICR mice, demonstrating that Aβ introduced to CSF is transported into aqueous humor.

In general, where you say central nervous system, please change to CSF for better clarity, as you are referring to soluble Aβ(1-42).

[Reply] Thank you very much for the comment. We applied the change in the revised manuscript. Also, we rewrote the entire abstract and introduction to tone down and make our hypothesis and results clearer.

*Abstract
• Line 25: “Based on the clinical evidence…”*
[Reply] Thank you for your considerate direction. We have deleted the sentence to clarify the conveying messages.

(Line 25) Based on the clinical evidences

*• Line 27: Please clarify what specifically is correlated; i.e. presence/concentration of Aβ in aqueous humor is positively correlated with the presence/concentration if Aβ in CSF.*
[Reply] We have revised the sentence as below;

(Line 27) the Aβ in the aqueous humor in AD models is directly correlated with the misfolding protein of the central nervous system.

(Line 28) Given that the human eye structurally resembles the brain and Aβ accumulation often observed in the ocular region of AD patients, in this study, we examined aqueous humor Aβ as another possible surrogate biomarker.

*• Line 31: Consider changing wording, i.e. “To investigate the correlation between Aβ levels in CSF and aqueous humor, we…”*
[Reply] We rephrased the sentence as below;

(Line 31) To investigate the direct correlation between Aβ levels between CSF and aqueous humor

(Line 30) using the acute Aβ-infused AD mouse model by injecting Aβ to the CSF in intracerebroventricular region of normal ICR mice, we investigated whether Aβ concentration in the aqueous humor in AD models is positively correlated with the concentration in the CSF.

*• Line 37: Consider changing conclusion wording, i.e. “Our results indicate that Aβ peptides are elevated in the aqueous humor in AD mice. In addition to this, acute elevation of Aβ peptides through injection into CSF can be detected in the aqueous humor, suggesting that*
[Reply] Thank you for the considerate suggestion. Combined with the conclusion from the 5XFAD mice, we revised the sentence as below;

(Line 37) Our results indicate that Aβ peptides detected in the aqueous humor can be used as a surrogate biomarker of perturbed Aβ levels in central nervous systems of AD.

(Line 35) Collectively, the synthetic Aβ injected into CSF immediately migrate to the aqueous humor, however, the age-dependently reducing pattern of Aβ levels in CSF and blood was not observed in the aqueous humor.

*Introduction
• Line 47: Other limitations to the amyloid-PET scan e.g. cost, exposure to radiation?*

[Reply] We rephrased the sentence as below;

(Line 47) The only limitation of the amyloid-PET scan is the difficulty to assess the diagnosis on subjects in preclinical stages of AD due to the lack of cognitive symptoms and voluntarily enrollments

(Line 50) The definitive diagnosis of AD has required detection of Aβ deposits in the brain either by biopsy, autopsy, or positron emission tomography along with signs of neurodegeneration.

*• Line 59: “The eye has been suggested…”*

[Reply] We removed the prepositional phrase in the sentence like below.

(Line 37) In many years, the eye has been suggested as a window of the brain

(Line 71) it is no surprise that the eye has been studied as a window of the brain.

*• Section beginning with Line 59 is generally unclear; please clarify why you have included information regarding POAG, i.e. do you mean that Aβ in aqueous has been found to be elevated in POAG, and may play a role in neurodegenerative diseases in the eye, and so measuring Aβ in aqueous humor may be useful for AD as well as other neurodegenerative diseases?*

[Reply] We agree that this sentence could make the readers confused. We have revised the sentence as below. The association between POAG and AD is described in the discussion section, instead of introduction.

(Line 59) In many years, the eye has been suggested as a window of the brain.

(Line 70) Given the shared functional and structural features of brain and ocular tissues, it is no surprise that the eye has been studied as a window of the brain.

*• Line 61: Reference 6 does not directly measure levels of Aβ in aqueous humor of patients, so does not support your statement.*

[Reply] Thank you for your critical observation. We have rewritten the manuscript focusing on the possibility of detecting Aβ in the aqueous humor samples. We hope that the edited section clarifies the meaning.

(Line 61) Aβ is detected at high levels in the aqueous humor of patients who suffer from open-angle glaucoma.

(Line 78) A previous study reported that the higher Aβ(1-40) level was detected in aqueous humor of AD patients.

*• Line 62: Please clarify logic for this sentence, your meaning is unclear. Reference 10 is a review paper that discusses the role of Aβ in AMD, and how changes in Aβ may be involved in the early stages of AMD pathogenesis. Do you mean that changes in Aβ levels in aqueous humor may also be seen in the early stages of AD pathogenesis?*

[Reply] Perhaps you meant the reference 9. We had regarded as POAG and AMD in the same context, having the retinal degeneration in that POAG ultimately induces retinal degeneration. General theory regarding the association between AD and glaucoma or AMD is revealed in the reference. Sivak JM. The aging eye: common degenerative mechanisms between the Alzheimer's brain and retinal disease. Invest Ophthalmol Vis Sci. 2013;54(1):871-80.[2]

To conform to your query, we have removed the reference and clarified the meaning in the revision as below.

(Line 63) The biochemical changes in the eye precedes the neurological symptoms, thus the sampling of aqueous humor usually takes place before the appearance of AD symptoms, enabling to achieve early-diagnosis of AD.

(Line 308) Since these ophthalmic biochemical changes occur earlier than the onset of AD, aqueous humor Aβ(1-42) can be analyzed in the preclinical stage of AD.

*• Line 72: “Given that human aqueous humor samples have been shown to contain Aβ…” this statement needs a reference*
[Reply] The reference is now added. Goldstein LE, Muffat JA, Cherny RA, Moir RD, Ericsson MH, Huang X, et al. Cytosolic beta-amyloid deposition and supranuclear cataracts in lenses from people with Alzheimer's disease. Lancet. 2003;361(9365):1258-65

(Line 72) Given that the aqueous humor samples contain Aβ.

(Line 78) A previous study reported that the higher Aβ(1-40) level was detected in aqueous humor of AD patients.

*Results*
*• Line 140: Should be “Results”*
[Reply] Thank you for your elaborate point. We have revised the result to results.

(Line 227) Result

(Line 164) Results

*• Line 150: You have stated that Aβ(1-42) was not found in the aqueous humour of WT mice, and you refer to Figure 1. However, Figure 1 shows that > 500 pg/mL of Aβ(1-42) was found in 12-month-old WT, and approx. 250 pg/mL of Aβ(1-42) was found in 24-month-old WT. In addition to this, you noted on line 125 that the analytical sensitivity of your sandwich-ELISA is < 1.0 pg/mL. Please clarify this discrepancy. Do you mean that Aβ(1-42) was present in significantly lower levels in WT compared to TG model?*
[Reply] Thank you for the comment. This data is close to the background noise level and to circumvent the confusing data, we have decided to delete the WT mice group. Also, endogenous Aβ(1-42) detection issue in the murine was previously reported. Teich AF, Patel M, Arancio O. A reliable way to detect endogenous murine beta-amyloid. PLOS One. 2013;8(2):e55647. [1]

*• Figure 1: Please state whether you have plotted means ± SD or means ± SEM in the figure caption, include the sample size as a legend and add brackets to indicate which two groups you are referring to for each p-value*
[Reply] Thank you for the critical insights. We have plotted means ± SEM in the figure. We have revised all figure captions including sample size.

*• For the data shown in Figure 1, you have used an F-test to determine the difference in Aβ(1-42) levels between WT and TG, which assumes both datasets are normally distributed, and have equal variances. Please include the statistical test for normality, and provide a table summarizing the numerical mean ± SD for each group*
[Reply] Thank you for the critical insights. We have plotted means ± SEM in the figure. We have displayed the standard deviation value and have done the normality test, and each analysis is summarized in S1 table.

*• Please include the F-value, and degrees of freedom, for each p-value you have given for Figure 1*
[Reply] We have included the F-value, the degrees of freedom, and each p-value in S1 table.

*• Line 153: Please perform a statistical analysis to determine whether levels of Aβ(1-42) are significantly different between TG 12 months and TG 24 months*
[Reply] In the revised manuscript, we changed our transgenic mice from APP/PS1 to 5XFAD to expand the study with both genders and two different age models per gender. Unpaired t-test was performed to analysis the difference between the adult and aged mice.

*• If all datasets are normally distributed with equal variance, consider doing a one-way ANOVA for all 4 datasets, and then perform a Tukey post hoc test to determine which means are significantly different from each other amongst all 4 groups*
[Reply] Thank you for the critical insights. We have revised all statistical analysis. All ANOVA data were determined to be Tukey post hoc test.

*• Line 154: Please change “subjects” to “in ageing TG mice”; also, please reword this sentence, the logic is not clear.*
[Reply] We made the correction.

(Line 154) it was reported that CSF Aβ(1-42) levels tend to decrease as subjects age and that inverse correlation of Aβ levels between CSF and blood samples upon aging in AD transgenic mouse models.

(Line 254) In the CSF, Aβ(1-42) concentration decreased as ages increased (**Fig 3C**). Plasma Aβ(1-42) level also showed a decreasing tendency along the ages (**Fig 3D**). In consistent with previous reports in AD-related transgenic mouse models, the decrease of Aβ(1-42) levels in CSF and plasma was reproduced in this study

*• Line 157: Please change “the eye” to “aqueous humor”.*
[Reply] We made the correction.

(Line 157) Thus, the age-dependent increase of the aqueous humor Aβ(1-42) may imply that the Aβ transport from the central nervous system to the eye is through blood.

(Line 218) Thus, the migration route of Aβ from the CSF to the aqueous humor is probably through the blood stream.

*• Line 187: In the caption for Figure 2, please change “central nervous system” to “CSF” and “eye” to “aqueous humor”*
[Reply] We made the correction.

(Line 187) To confirm that Aβ migrates from central nervous system to the eye,

*• Line 195: please change “central nervous system” to “CSF” and “eye” to “aqueous humor”*
[Reply] We made the correction.

(Line 195) Interim results support our hypothesis that Aβ(1-42) can transport from the central nervous system to the eye in AD models.

(Line 195) Interim results support our hypothesis that Aβ(1-42) can transport from CSF to the aqueous humor in the mouse models.

*• Line 209: please change to “…that acute changes in the concentration of Aβ(1-42) in the CSF…”*
[Reply] Thank you for the critical insights. We have revised the sentence.

(Line 209) the concentration perturbation of Aβ(1-42) in the central nervous system can be reflected in the aqueous humor.

(Line 207) acute changes in the concentration of Aβ(1-42) in the CSF can be reflected in the aqueous humor.

*• Line 212: This sentence needs a citation*
[Reply] Thank you for the critical insights. We added citation to this sentence.

Kanekiyo T, Bu G. The low-density lipoprotein receptor-related protein 1 and amyloid-beta clearance in Alzheimer's disease. Front Aging Neurosci. 2014;6:93.

*• Please perform a correlation between levels of Aβ(1-42) in CSF and Aβ(1-42) in aqueous humor to show how strongly these are related, include the resulting r2 value and statistics to support statements regarding the “significant correlation” e.g. line 222, and generate a figure showing this data*
[Reply] Thank you for your elaborate point. We added figure 2D in the results section to analyze the correlation between levels of Aβ(1-42) in CSF and aqueous humor.

*• Please include the sample size for each time point as a legend, and state in the figure caption whether you have plotted mean ± SD or mean ± SEM*
[Reply] Thank you for the critical insights. We have included the sample size for each time point as a legend. Mean ± SEM statistical analysis was performed in the figures.

*• In Figure 2, the level of Aβ(1-42) in aqueous humor after 4 nmole ICV injection was approx. 6000 pg/mL, however in Figure 3, the level of Aβ(1-42) in aqueous humor after 4 nmole ICV injection appears to be half of this, at approx. 3000 pg/mL. Can you comment on why you think this may be the case, as the protocol was the same?*
[Reply] Thank you for the important comment. Since the original data is too minimal to be differentiated, dilution factor of the samples has a great impact on the results. We initially made aqueous humor diluted to 1/200 when designing time-dependent aqueous humor exam. But, on the following experiment, we had to produce aqueous humor diluted to 1/100, since the ELISA value was not detectable in the low concentration group when diluted to 1/200. Therefore, two peaks seemed to be different between the graphs.

*• For Figure 3, you state you have plotted mean ± SEM, however it appears you have used box plots. Please clarify what you have plotted. If they are box plots, this is a good indication of the spread of your data, but please also provide mean ± SD values in a table.*
[Reply] Thank you for the critical directions. As using box plots, we have adopted mean ± SEM*.* The standard deviation of each variable is now provided in the S1 Table.

*• You have used a one-way ANOVA to determine whether the levels of Aβ(1-42) in CSF, aqueous humor and plasma are significantly different following different injection concentrations, which assumes that datasets are normally distributed and have equal variances. Please include the statistical tests for normality.*
[Reply] All results were analyzed according to D'Agostino & Pearson normality test, but the data were not analyzed due to the small number of individuals.

*• For the data provided in Figure 3, please report the F-values and degrees of freedom from your one-way ANOVA. Also, please clarify which pairwise comparison was significantly different, either on the figure with brackets, or in a table.*
[Reply] Thank you for the critical insights. We have included the F-value, the degrees of freedom, and each p-value in S1 table.

*• Please include the data for level of Aβ(1-42) in plasma when 4 nmole of Aβ(1-42) was injected, or provide details regarding why this data point was excluded from Figure 3D*
[Reply] Thank you for your elaborate point. We newly analyzed the Aβ(1-42) level in plasma of each ICV injection (0.5, 1 , 2, and 4 nmol) in figure 2.

*Discussion
• Line 228 should read “Aβ levels in CSF and aqueous humor”*

[Reply] Thank you. We edited the manuscript to remove the sentence containing the phrase.

(Line 228) Our study aimed to investigate the link between the Aβ levels in CSF and aqueous humor.

*• Line 230: Is this statement accurate? To my understanding, although ICR mouse does not specifically express human APP, this mouse model still expresses endogenous murine APP which may produce Aβ which may be detected by ELISA. In addition to this, your WT control had detectable levels of Aβ in aqueous. Better evidence of the movement of Aβ from CSF to aqueous humor would be the change in Aβ concentration over time, as well as the significant changes in Aβ levels in aqueous when you alter injected CSF Aβ concentrations.*

[Reply] In fact, the ELISA kit (#KHB3544) used in the experiment is only validated in human Aβ*(*1-42), hence how the positive value can be drawn is not unraveled. The wild type group as seen in the reference below, also showed a positive value when using same product. Teich AF, Patel M, Arancio O. A reliable way to detect endogenous murine beta-amyloid. PLoS One. 2013;8(2):e55647.

*• Line 239: You may also wish to add, to further support your argument, that it is known that aqueous humor is composed of blood plasma (REF), and it is believed that aqueous humor proteins are derived from blood plasma, rather than synthesised locally inside the eye (REF).*

[Reply] Thank you for your support. We have rewritten the discussion part and a reference is now added.

Goel M, Picciani RG, Lee RK, Bhattacharya SK. Aqueous humor dynamics: a review. Open Ophthalmol J. 2010;4:52-9.

(Line 239) The aqueous humor is also secreted from the ciliary body that contains blood vessels

(Line 286) Aqueous humor is secreted from the ciliary body, the circulation of which produces aqueous humor by blood ultrafiltration

*• Line 240: “Thus, it is possible…”*
[Reply] To comply the reviewer’s comment, we underwent the intravenous injection to look in the way of *Aβ*. As a result, we redrafted the sentence with more firming tone.

(Line 240) Thus, it is predictable that the Aβ monomer passes blood-brain barrier and ultimately enters the

aqueous humor

(Line 288) We concluded that the Aβ monomer passes blood-brain barrier and ultimately enters the aqueous humor.

*• Line 242: Consider discussing results in the order that they have been presented e.g. WT vs. TG, then ICV injection to ICR mice*

[Reply] We made this correction. Thank you.

*• Line 245: “known to precede other processes like the…”*
[Reply] We deleted the sentence in the revision to convey our meaning concisely.

*References
• Line 280 should be “References”*
[Reply] We made this correction.

(Line 280) Reference

(Line 340) References

*Minor Comments*
*• In my version, the figures look pixelated (e.g. text is hard to read in Figure 3). Please check the resolution of all figures.*
[Reply] Thank you for your elaborate point. All figures and tables were replaced with 300 dpi resolution ones.

**Reference**

1.	Teich AF, Patel M, Arancio O. A reliable way to detect endogenous murine beta-amyloid. PLoS One. 2013;8(2):e55647.

2.	Sivak JM. The aging eye: common degenerative mechanisms between the Alzheimer's brain and retinal disease. Invest Ophthalmol Vis Sci. 2013;54(1):871-80.
